# Autoimmune Processes and Chronic Inflammation as Independent Risk Factors for Metabolic Complications in Women with Polycystic Ovary Syndrome

**DOI:** 10.3390/metabo15030141

**Published:** 2025-02-20

**Authors:** Katarzyna Suchta, Natalia Zeber-Lubecka, Monika Grymowicz, Roman Smolarczyk, Maria Kulecka, Ewa E. Hennig

**Affiliations:** 1 Department of Gynecological Endocrinology, Medical University of Warsaw, 2 Karowa Street, 00-315 Warsaw, Poland; 2 Department of Gastroenterology, Hepatology and Clinical Oncology, Centre of Postgraduate Medical Education, 5 Roentgena Street, 02-781 Warsaw, Poland; 3 Department of Genetics, Maria Sklodowska-Curie National Research Institute of Oncology, 5 Roentgena Street, 02-781 Warsaw, Poland

**Keywords:** polycystic ovary syndrome, chronic inflammation, autoimmune thyroid disease, metabolism, obesity, lipid profile, body mass index

## Abstract

Background: Polycystic ovary syndrome (PCOS) and autoimmune thyroid disease (AITD) have a high prevalence in women of reproductive age. PCOS can lead to long-term adverse health effects such as obesity, diabetes, and increased metabolic and cardiovascular risk. Although it is known that subclinical and clinical hypothyroidism may also worsen body mass index (BMI), lipid profile, and metabolic risk, there are few studies on the impact of elevated thyroid autoantibodies alone and associated chronic inflammation on metabolic complications in women with PCOS. The main aim of the study was to assess the prevalence of AITD among Polish women with PCOS and the metabolic impact of the co-occurrence of both diseases in euthyroid individuals. The additional aim was a review of the literature on the prevalence of co-occurrence of PCOS and AITD and the metabolic consequences of this condition. Methods: A total of 424 women aged 16–46 years were recruited into the study—230 women diagnosed with PCOS and 194 women diagnosed with PCOS and co-occurrence of euthyroid AITD. Before participating in the study, patients signed a written informed consent. The study was approved by the local ethics committee. Statistical analysis was performed using IBM SPSS Statistics (v.25). A mini-review of the literature was performed using the PubMed database. Results: Women with co-occurrence of PCOS and euthyroid AITD had statistically significantly higher serum levels of total cholesterol (189.57 mg/dL vs. 180.16 mg/dL; *p* = 0.005; d Cohen’s = −0.278), LDL-cholesterol (109.80 mg/dL vs. 102.01 mg/dL; *p* = 0.009; d Cohen’s = −0.256), and triglycerides (107.77 mg/dL vs. 96.82 mg/dL; *p* = 0.027; d Cohen’s = −0.219) compared to women with PCOS. The difference was observed regardless of body weight. BMI was also statistically significantly higher in the PCOS-AITD group (27.55 kg/m^2^ vs. 25.46 kg/m^2^; *p* = 0.003; d Cohen’s = −0.319), as was the prevalence of obesity (32.5% vs. 20.7%; Chi-square = 7.956; *p* = 0.047). The mini-review of the literature did not find many studies evaluating the impact of thyroid autoantibodies on metabolic outcomes in PCOS euthyroid women, and the data are still inconclusive. Conclusions: The presence of elevated serum concentrations of thyroid autoantibodies in euthyroid women with PCOS increases the risk of obesity and metabolic consequences. It is observed even in euthyroid and non-obese individuals. Consequently, the cardiovascular risk in these women may be higher than in PCOS women without elevated thyroid autoantibodies. It is important to assess thyroid autoantibodies in all women with PCOS. In euthyroid PCOS women with co-occurrence of elevated serum levels of thyroid autoantibodies, it is crucial to pay more attention to maintaining an appropriate body mass index. There is an urgent need for further studies in large groups of women assessing the impact of elevated thyroid autoantibodies alone on metabolic outcomes in euthyroid women with PCOS to confirm and clarify the results.

## 1. Introduction

Polycystic ovary syndrome (PCOS) is one of the most common endocrine disorders in women of reproductive age, with a point prevalence of up to 15% worldwide [1]. Characterized by the Rotterdam criteria, PCOS has a significant impact on women’s physical and mental health. PCOS is a heterogeneous disorder in which four phenotypes can be observed. These phenotypes are characterized by variations in the presence or absence of the Rotterdam criteria (hyperandrogenemia, ovulatory dysfunction, and specific ovarian ultrasound morphology). Women with different phenotypes of the syndrome are at different risks of certain health outcomes [2]. Women with PCOS are generally at higher risk of metabolic outcomes such as obesity, impaired glucose tolerance, and type 2 diabetes, but they also have a higher cardiovascular risk and a higher prevalence of infertility, miscarriage, and pregnancy complications. In addition to physical disturbances, women with PCOS are also at higher risk of mental disorders such as anxiety, depression, lower quality of life, lower self-esteem, and sexual dysfunction [3,4,5]. The etiology and pathogenesis of PCOS remain unclear. 

Many researchers highlight the fact of a higher prevalence of thyroid diseases such as autoimmune thyroid disease (AITD) and subclinical or clinical hypothyroidism in women with PCOS. According to studies, the prevalence of AITD in women with PCOS varies from 18% to 40% and depends on the ethnicity of the women and the diagnostic criteria of PCOS used at diagnosis [6]. AITD is defined as the presence of elevated thyroid peroxidase or thyroglobulin autoantibodies (TPO-Ab, TG-Ab). The most common clinical presentation of AITD is Hashimoto’s thyroiditis (HT), where elevated TPO-Ab and TG-Ab are associated with decreased thyroid echogenicity on ultrasound. HT is the most common autoimmune disease affecting 5 to 20% of women of reproductive age and results in chronic inflammation of the thyroid gland [7]. Subclinical or clinical hypothyroidism may be observed over the course of the disease. Temporary episodes of hyperthyroidism may also occur, especially at the onset of the disease.

The diagnosis of hypothyroidism in women is associated with an increased risk of metabolic and reproductive disorders [8,9,10,11,12,13].

It has been observed that the clinical manifestations of both conditions—PCOS and hypothyroidism—can be similar, such as menstrual irregularities, inappropriate lipid profile, obesity, or insulin resistance. There are studies in the literature suggesting that the co-occurrence of PCOS and subclinical or clinical hypothyroidism may increase the risk of metabolic disorders [6]. Some previous research has emphasized the role of hypothyroidism and altered thyrotropin (TSH) levels in the long-term adverse health effects of PCOS [6,14,15].

Although it is known that hypothyroidism has a negative impact on metabolic health, to the best of the authors’ knowledge, there are currently very few studies investigating the role of elevated thyroid autoantibodies in euthyroid individuals before the onset of hypothyroidism and their impact on metabolic status. In women with PCOS, there is even less literature [14,15,16,17]. In addition, there is still no comprehensive and reliable study based on a large group of patients in the Polish population with PCOS, investigating the impact of TPO-Ab and TG-Ab alone on obesity, metabolic, and cardiovascular risk.

Considering that both PCOS and AITD have a high prevalence in young women, that the prevalence of AITD is higher in women with PCOS, and that both conditions can have an impact on health status and long-term quality of life, in our study, we decided to investigate whether the co-occurrence of autoimmune thyroiditis in euthyroid patients before the onset of hypothyroidism worsens health status and aggravates obesity, metabolic and cardiovascular risk in women with PCOS.

## 2. Materials and Methods

### 2.1. Ethics Statement

The study was approved by the Ethics Committee of the Medical University of Warsaw (No: KB/200/2015). All patients included in the study were Polish Caucasians attending the endocrinological gynecology clinic at the Department of Endocrinology and Gynaecology, Medical University of Warsaw. Before participating in the study, patients gave written informed consent.

### 2.2. Study Population

A total of 424 women diagnosed with PCOS, aged 16 to 46 years, were recruited for this study. The study group was screened for AITD. Participants’ age, body mass index (BMI), TSH, free thyroxine (FT4), fasting glucose and insulin, glucose after 2 h of oral glucose tolerance test (OGTT) using 75 g glucose, insulin after 1 h and 2 h of OGTT using 75 g glucose, and lipid profile were also assessed. The ranges of standard concentrations of the parameters assessed are shown in Table S1 in the Supplementary Materials. The study group was then divided into two subgroups:

- Group 1—women diagnosed with PCOS (230 women) [PCOS],
- Group 2—women diagnosed with PCOS and euthyroid AITD (194 women) [PCOS-AITD].

#### 2.2.1. PCOS Assessment

The diagnosis of PCOS was made according to the Rotterdam criteria, modified by international consensus of the American Society for Reproductive Medicine (ASRM), the Endocrine Society (ENDO), the European Society of Endocrinology (ESE) and the European Society of Human Reproduction and Embryology (ESHRE), which include clinical and/or biochemical hyperandrogenism; anovulatory menstrual cycles and/or infrequent ovulations; and characteristic ovarian structure on ultrasound [18].

Clinical hyperandrogenism was defined as hirsutism, acne, and/or alopecia. The degree of hirsutism was assessed using the Ferriman-Gallwey score. A score of 4 or more confirmed hirsutism. Biochemical hyperandrogenism consists of elevated levels of androgens (testosterone and/or androstenedione).

Anovulation was confirmed by oligomenorrhea and serum progesterone levels on days 22 to 24 of the cycle. A level of 3 ng/mL or less confirmed an anovulatory cycle. Oligomenorrhea was defined as a menstrual cycle lasting more than 35 days, and secondary amenorrhea was defined as the absence of menstrual bleeding for more than 6 months.

A pelvic ultrasound was performed using an Aloka 7 alpha device (Hitachi-Aloka Medical America Inc., Wallingford, CT, USA) to assess ovarian morphology. Polycystic ovary morphology (PCOM) was defined as the presence of follicle number per ovary (FNPO) ≥ 20 in at least one ovary or follicle number per section (FNPS) ≥ 10 in at least one ovary and/or ovarian volume > 10 mL in the absence of confounding pathology.

Serum total testosterone, TSH, FT4, TPO-Ab, TG-Ab, and insulin levels were measured by the immunochemical method—chemiluminescence on the Alinity i2000SR System (Abbott Ireland, Abbott Laboratories Poland, Warsaw, Poland). The serum concentration of androstenedione was tested by the chemiluminescent immunoassay technique by Immulite 2000XPi (Siemens Healthcare Poland, Warsaw, Poland). Serum glucose concentration was tested by enzymatic method with hexokinase on the Cobas INTEGRA 400 Plus System (Roche Diagnostisc Poland, Warsaw, Poland). Serum concentration of triglycerides, total cholesterol, and high-density lipoprotein cholesterol (HDL cholesterol) were tested by the enzymatic colorimetric method on the Cobas INTEGRA 400 Plus System (Roche Diagnostisc Poland, Warsaw, Poland). Serum low-density lipoprotein cholesterol (LDL cholesterol) level was measured by computational method.

Exclusion criteria were patient refusal to participate in the study and a diagnosis of hyperandrogenism other than PCOS, such as non-classical adrenal hyperplasia, androgen-secreting tumor, Cushing’s syndrome, hyperprolactinemia, or non-euthyroid thyroid dysfunction. An additional exclusion criterion was recent pregnancy, use of oral contraceptives, glucocorticoids, biguanides, or glucagon-like peptide 1 (GLP-1) receptor agonists for up to six months prior to enrollment, as all these medications can affect hormonal and biochemical parameters.

#### 2.2.2. AITD Assessment

The diagnosis of AITD included an elevated serum level of thyroid peroxidase antibodies (TPO-Ab) or thyroglobulin antibodies (TG-Ab). The diagnosis of HT included at least an elevated serum level of TPO-Ab or TG-Ab and decreased thyroid echogenicity on ultrasound. Thyroid echogenicity was assessed in both thyroid lobes and in the muscles surrounding the neck. Hypoechogenicity was assessed by comparing the distribution of echoes in the thyroid parenchyma with those in the surrounding neck muscles. In patients with a diagnosis of HT at the time of enrollment and blood sampling, all patients were euthyroid. Exclusion criteria were patient refusal to participate in the study and subclinical or clinical hypothyroidism.

### 2.3. Statistical Analysis

Statistical analysis was conducted using IBM SPSS Statistics for Windows, version 29 (IBM Corp., Armonk, NY, USA). The normality of data was checked by the Shapiro–Wilk test. The medians were calculated. The independent-samples *t*-test, also known as the unpaired *t*-test, was used to determine whether there is a difference between two independent, unrelated groups. The Chi-square test of independence was used to determine if there is a significant relationship between two nominal (categorical) variables. A *p*-value of <0.05 was considered significant. To assess the size of the effect, Cohen’s d was used, and it was classified as small (d = 0.2–0.3), medium (d = 0.5–0.8), and large (d > 0.8). The Spearman rank–order correlation coefficient (Spearman’s correlation, for short) was used to measure the strength and direction of association that exists between two variables.

### 2.4. Mini-Review of the Literature

The literature was reviewed using the PubMed database.

To evaluate recent studies on the co-occurrence of AITD in women with PCOS, the combination of the words “AITD”, “autoimmune thyroid disease”, “HT”, “Hashimoto thyroiditis”, “AIT”, “autoimmune thyroiditis”, “PCOS”, and “polycystic ovary syndrome” was used and the search scope was set to the years 2013–2024.

To evaluate recent research on the metabolic consequences of the co-occurrence of PCOS and AITD, combinations of the words “PCOS”, “polycystic ovary syndrome”, “AITD”, “autoimmune thyroid disease”, “autoimmune thyroid disease”, “AIT”, “autoimmune thyroiditis”, “HT”, “Hashimoto’s thyroiditis”, “metabolic risk”, “metabolic consequences”, “lipid profile”, “BMI”, “body mass index”, “body mass index”, “IR”, “insulin resistance”, and “diabetes” were used and the search scope was set to the years 2013–2024.

## 3. Results

### 3.1. Intergroup Comparison

Table 1 presents the comparison of basal clinical and biochemical parameters of the PCOS group and euthyroid PCOS-AITD group.

The median age for the PCOS group was 25.22 years compared to the PCOS-AITD group, where the median age was 28.02 years (*p* < 0.001; d Cohen’s = −0.714).

There was also a statistically significant difference between the BMI levels of the groups evaluated. The median BMI in the PCOS group was 25.46 compared to the PCOS-AITD group, where the median BMI was higher (27.55; *p* = 0.003; d Cohen’s = −0.319).

There was a statistically significant difference in TSH between the groups. The median TSH level was higher in the PCOS-AITD group compared to the PCOS group, but remained within the reference range in both groups [2.12 µIU/mL vs. 1.62 µIU/mL; (*p* = 0.010; d Cohen’s = −0.272)].

There were no statistically significant differences in mean FT4 levels between the study groups.

There also appeared to be statistically significant differences in the mean levels of TPO-Ab or TG-Ab, which were higher in the PCOS-AITD group than in the PCOS group [TPO-Ab—289.19 IU/mL vs. 0.48 IU/mL; (*p* < 0.001; d Cohen’s = −0.837); TG-Ab—140.73 IU/mL vs. 1.7 IU/mL; (*p* = 0.003; d Cohen’s = −0.319)].

Among the metabolic biochemical parameters, there were no statistically significant differences in the mean values of glucose at any time of the OGTT using 75 g glucose; however, the mean value of fasting glucose was higher in the PCOS group compared to the PCOS-AITD group [91.98 mg% vs. 89.49 mg%; (*p* = 0.519)].

In comparison, the mean value of insulin at any time of the OGTT using 75 g glucose was higher in the PCOS-AITD group than in the PCOS group, but it was not statistically significant [insulin 0 min—8.16 µU/L vs. 7.71 µU/L, (*p* = 0.332); insulin 60 min—69.55 µU/L vs. 61.96 µU/L, (*p* = 0.085); insulin 120 min—66.42 µU/L vs. 62.06 µU/L, (*p* = 0.442)].

Homeostatic Model Assessment for Insulin Resistance (HOMA-IR) was also higher in the PCOS-AITD group compared to the PCOS group, but it was not statistically significant [1.82 vs. 1.7, (*p* = 0.259)].

In our study, we found statistically significant differences in most lipid parameters between the groups. The mean value of total cholesterol was higher in the PCOS-AITD group than in the PCOS group [189.57 mg/dL vs. 180.16 mg/dL; (*p* = 0.005; d Co-hen’s = −0.278)]. Mean low-density lipoprotein cholesterol (LDL Chol) was also higher in the PCOS-AITD group than in the PCOS group [109.8 mg/dL vs. 102.01 mg/dL; (*p* = 0.009; d Cohen’s = −0.256)].

The mean level of high-density lipoprotein cholesterol (HDL Chol) was also higher in the PCOS-AITD group than in the PCOS group, but it was not statistically significant [59.51 mg/dL vs. 58.2 mg/dL; (*p* = 0.440)]. The mean value of triglycerides (TG) was higher in the PCOS-AITD group than in the PCOS group, and it was statistically significant [107.77 mg/dL vs. 96.82 mg/dL; (*p* = 0.027; d Cohen’s = −0.219)].

### 3.2. Link Between Thyroid Autoimmunity and Metabolic Parameters

Table 2 and Table 3 present the main and crucial findings concerning the relationship between autoimmunity and metabolic parameters in euthyroid individuals.

In our research, we found a statistically significant relationship between autoimmunity, expressed as thyroid autoantibody levels (TPO-Ab and TG-Ab), and BMI (Chi-square = 7.956; *p* = 0.047), which is presented in Table 2. Among women in the PCOS-AITD group, the prevalence of obese women was statistically significantly higher than in the PCOS group (32.5% vs. 20.7%). In comparison, the prevalence of normal weight was statistically significantly lower in the PCOS-AITD group than in the PCOS group (40.2% vs. 51.8%).

There was also a statistically significant positive correlation between autoimmunity (TPO-Ab and TG-Ab) and BMI. For TPO-Ab Rho = 0.122 with *p*-value = 0.016 and for TG-Ab Rho = 0.159 with *p*-value = 0.002 (Table 3).

In our study, there was a statistically significant positive correlation between autoimmunity (TPO-Ab and TG-Ab) and carbohydrate metabolism, which is presented in Table 3 and includes:

- fasting glucose—for TG-Ab Rho = 0.159 with *p*-value = 0.002,
- fasting insulin—for TPO-Ab Rho = 0.171 with *p*-value ≤ 0.001; for TG-Ab Rho = 0.110 with *p*-value = 0.023,
- insulin serum level after 120 min in oral glucose tolerance test—for TPO-Ab Rho = 0.180 with *p*-value ≤ 0.001,
- HOMA-IR—for TPO-Ab Rho = 0.170 with *p*-value ≤ 0.001; for TG-Ab Rho = 0.134 with *p*-value = 0.006.

The results of our research also show a statistically significant positive correlation between autoimmunity (TG-Ab) and lipid profile, which is presented in Table 3 and includes

- total cholesterol—for TG-Ab Rho = 0.141 with *p*-value = 0.004,
- LDL cholesterol—for TG-Ab Rho = 0.143 with *p*-value = 0.003,
- TG (triglycerides)—for TG-Ab Rho = 0.119 with *p*-value = 0.015.

All the positive correlations between autoimmunity and metabolic parameters were observed regardless of body weight.

## 4. Discussion

### 4.1. Co-Occurrence of PCOS and AITD

Autoimmune thyroid disease (AITD), also known as Hashimoto’s thyroiditis (HT) or chronic lymphocytic thyroiditis, is considered one of the most common causes of hypothyroidism and is one of the most common autoimmune diseases in young women. The immanent element of the disease is the presence of inflammatory cells infiltrating the thyroid gland as well as the presence of thyroid autoantibodies—anti-thyroid peroxidase (the most characteristic) and/or anti-thyroglobulin. The presence of thyroid autoantibodies leads to activation of the immune system and complement/antibody-dependent cellular cytotoxicity. The result of this process is the destruction of thyroid cells and, over time, the development of hypothyroidism [8].

There are few studies that have investigated the co-occurrence of PCOS and AITD in women with euthyroidism and the associated health outcomes. The results of these investigations remain inconsistent and unclear and are presented in Table 4 [6,19,20,21,22,23,24].

Du D. and Li X.; Karaköse M. et al.; Romitti M. et al.; Ho CW. et al.; Sharma M. et al. have suggested and confirmed an increased prevalence of AITD in women with PCOS and a possible association between the two conditions [6,19,20,22,23].

In our study, the prevalence of euthyroid AITD in women with PCOS was 45%, confirming the conclusions of the majority of cited authors about the increased incidence of AITD in people with PCOS. 

However, Duran C. et al. and Kim JJ. et al. did not show such a relationship [21,24]. It is worth noting that the study groups in these authors’ research were not as large. There may also be an effect of the geographical region and ethnicity of the study groups, which may influence the results.

### 4.2. AITD as a Chronic Inflammation Disease That Involve Immune System

The essence of AITD is the presence of thyroid autoantibodies (TPO-Ab and/or TG-Ab) that chronically infiltrate the thyroid gland. As a result of the long-term influence of TPO-Ab as a competitive inhibitor of TPO action, hypothyroidism develops. TPO-Ab also has the ability to induce complement activation and immune responses, destroy thyrocytes, and induce oxidative stress [25]. TPO-Ab usually belongs to immunoglobulin G1 (IgG1), but in lower prevalence, it may also belong to IgG2, IgG3, IgG4, and IgA. In women with AITD, TPO-Ab is produced by B cells as a result of the infiltration of the thyroid by autoreactive CD4+ T lymphocytes [26]. There is much research that confirms that thyroid autoantibodies have not only local but systemic influence on altering the immune system, which can have global consequences on the organism [16,26,27,28,29,30,31,32,33,34]. The presence of thyroid autoantibodies leads to changes in the activity of NK cells, B cells, and T helper cells. Elevated levels of TPO-Ab cause the induction of cytotoxic lymphocyte and macrophage activity and decreased prevention of Th1 cell differentiation. Consequently, these changes increase the Th1/Th2 ratio. As a result of these changes, impairment of immunological tolerance may occur, promoting an inflammatory microenvironment in the organism. At the same time, changes in NK cell activity lead to increased cytotoxicity of these cells. On the other hand, altered B cell activity leads to increased synthesis of non-organ-specific autoantibodies [16,26,27,28,29,30,31,32,33,34]. As an autoimmune disease, AITD often co-occurs with other autoimmune diseases, such as systemic lupus erythematosus or antiphospholipid antibody syndrome [35]. In such situations, there is an increasing concentration of non-specific autoantibodies produced by polyclonal activated B lymphocytes. These conditions exacerbate systemic autoimmunity and cross-reactivity with various antigens [36].

### 4.3. Potential Common Mechanisms of PCOS and AITD—Autoimmune Processes and Chronic Inflammation

In recent years, a growing body of research has highlighted the role of chronic inflammation, autoimmune disease, and oxidative stress in the etiology and pathogenesis of PCOS. 

Altered immune cell activity has been observed in women with PCOS. In PCOS patients, there were more macrophages and pro-inflammatory factors in adipose tissue [37]. It is also observed that PCOS in women is associated with increased neutrophils and neutrophil-to-lymphocyte ratio. This observation supports the statement that PCOS is associated with chronic low-grade inflammation [38]. In addition, a reduction in the percentage of dendritic cells has been observed in PCOS patients, as well as a disturbance in the associated cytokines. According to several authors, this may lead to disharmony in the immunological environment of the ovarian follicles [39,40].

Women with PCOS have a higher prevalence of overweight and obesity. Adipose tissue is a mediator of the chronic inflammatory process in these women, but it also influences the immune response [41]. In adipocytes, different immune cells can be observed—for example, neutrophils, M1 macrophages, or T cells [42]. Macrophages from adipose tissue play an important role in participating in systemic inflammatory responses by secreting adipose-inflammatory cytokines such as TNF-α, IL-6, and IL-1β. Another player in the immune response is leptin, which can stimulate Th1 cells to secrete excessive amounts of IFN-γ and lead to an exacerbation of the inflammatory response [41,42].

There are also studies that attempt to explain the phenomenon of the frequent co-occurrence of PCOS and AITD, and to answer the question of possible common mechanisms linking these two conditions. The potential cause of AITD in women with PCOS appears to be related to hyperandrogenism. Women with PCOS have an increased frequency of gonadotropin-releasing hormone (GnRH) and luteinizing hormone (LH) pulses, which can lead to elevated androgen levels [43]. High androgen levels can increase T-suppressor cell activity, but also promote Th1 responses. A higher prevalence of Th1 responses is also observed in AITD patients. Th1-mediated autoimmunity leads to thyroid cytolysis and hypothyroidism [20].

Women with PCOS have reduced levels of progesterone secretion, causing an imbalance between progesterone and estrogen levels. Estrogens increase the expression of IL-6 in T cells, and if at the same time the level of progesterone suppression is not sufficient, this can lead to overstimulation of the immune system. This imbalance in progesterone and estrogen levels makes PCOS patients more susceptible to autoimmune diseases [44].

### 4.4. Metabolic Effects of Co-Occurrence of PCOS and AITD

In Table 5, we present the results of a mini-review of the literature on the impact of AITD on metabolic consequences in PCOS women.

It is known that women with PCOS alone are at greater risk of metabolic consequences such as altered lipid profile or type 2 diabetes, especially if PCOS is associated with obesity. Some of these lead to metabolic syndrome and increased cardiovascular risk. In addition, it is known that patients with metabolic syndrome may have a clinical presentation that is similar to PCOS criteria. It is, therefore, important to exclude such individuals when making the diagnosis of PCOS [45]. In our study, we have shown that the co-occurrence of elevated thyroid autoantibodies in non-obese PCOS women with euthyroidism leads to an increased risk of the above-mentioned metabolic conditions. Elevation of aTPO and aTG is an autoimmune process that alters the immune system and increases chronic low-grade inflammation. This correlation is observed in euthyroid patients who are at a stage of the disease when hypothyroidism has not yet occurred or the thyroid function is well balanced by adequate levothyroxine supplementation. Remarkably, it is also observed in non-obese euthyroid women with elevated thyroid autoantibodies. Thus, it seems that thyroid autoantibodies alone (especially TPO-Ab) are factors that worsen metabolic prognosis regardless of BMI.

In our study, PCOS women with co-occurrence of elevated thyroid autoantibodies had an impaired lipid profile (higher total cholesterol, LDL-cholesterol, and triglyceride serum levels), higher body mass index, and higher prevalence of obesity compared to the PCOS group with normal thyroid autoantibody levels. Furthermore, an impaired lipid profile was also present in euthyroid PCOS patients with normal body mass index who had elevated thyroid autoantibody levels.

In the literature, there is some research exploring TSH variability and its influence on metabolism, but the impact of autoantibodies alone in euthyroid patients is rarely studied by researchers.

Romitti M. et al. [6] showed in their study that subclinical hypothyroidism in PCOS women with elevated thyroid autoantibodies correlates with mild changes in serum lipids as well as mild changes in HOMA-IR. The authors report no differences in BMI between PCOS and PCOS-AITD patients. They also did not confirm a correlation between AITD and BMI in PCOS women. Compared to our study, it does not assess the impact of elevated thyroid autoantibodies on the metabolic state in euthyroid individuals. Therefore, the results of this study can only be applied to non-euthyroid PCOS women. It shows the effect of TSH variability on metabolic consequences rather than the elevation of thyroid autoantibodies.

There is a meta-analysis in the literature on the effect of the co-occurrence of AITD in PCOS women on BMI, but it does not show an additional negative effect on BMI. No statistically significant difference in BMI was found between the PCOS and PCOS-AITD groups [6].

Cenlin J et al. also found no difference in body mass index between the PCOS-AITD and PCOS groups [15].

The conclusions of the above meta-analysis are incompatible with our results, where we showed that the co-occurrence of AITD in PCOS women worsens the risk of obesity and that the median BMI in the PCOS-AITD group was statistically significantly higher compared with the PCOS group. We can, therefore, hypothesize that the presence of elevated thyroid autoantibodies is an additional risk factor for obesity in PCOS women.

Ulrich J. et al. [17] showed in their research, similar to us, that the presence of elevated thyroid autoantibodies in euthyroid PCOS patients is associated with a higher body mass index (on average by 2 kg/m^2^). The authors also found that TSH levels were higher in patients with a higher BMI, although all patients remained euthyroid. The results of our study are similar to those of Ulrich J et al. We showed that in PCOS women with elevated thyroid autoantibodies, the prevalence of obesity was higher compared to PCOS without AITD—32.5% vs. 20.7% with an average BMI 2.09 kg/m^2^ higher. As a consequence, we also observed higher TSH levels in the PCOS-AITD group than in the PCOS group, although all patients were euthyroid—2.12 µIU/mL vs. 1.62 µIU/mL, with standard deviations of 2.62 vs. 0.66.

Ho CW. et al. [20] in their cohort study indicate a higher prevalence of diabetes, hyperlipidaemia, and coronary heart disease in the PCOS-AITD group compared to the PCOS group. However, this study has some limitations. The authors recruited both Hashimoto’s and Graves’ patients as AITD patients, so we cannot evaluate the effect of aTPO and aTG levels on metabolic risk. We also do not know the hormonal status of the participants, so we do not know if they were euthyroid, hypothyroid, or hyperthyroid. In addition, the main aim was to assess the prevalence of PCOS in AITD patients, and such a methodology is different from the aim of our study. All these facts mean that the study by Ho CW et al. is not comparable and reliable for assessing the effect of aTPO and aTG levels alone on metabolic outcomes.

There are not many studies in the literature assessing the impact of elevated thyroid autoantibodies in women with PCOS on cardiovascular risk, and data on subclinical and clinical hypothyroidism remain inconsistent.

Ho CW. et al. showed in their observational study that the co-occurrence of AITD and PCOS in patients with both conditions increases the cardiovascular risk approximately six-fold compared to patients with AITD alone [20].

On the contrary, other studies have not confirmed this conclusion. Collet et al., in their study, show no difference in cardiovascular mortality between the AITD group and the non-AITD group, although the subgroup of PCOS women was not analysed by the author [46]. Gawron et al. also indicated that the metabolic and so-called cardiometabolic risk show no difference between the PCOS group and patients with PCOS and AITD or subclinical hypothyroidism [47]. Because of the lack of consistent conclusions on this issue, we believe there is a need for further studies on this topic, conducted in a large population with appropriate and strict inclusion and exclusion criteria. Cardiovascular risk depends on metabolic factors such as BMI, central obesity, and lipid profile. These metabolic consequences in women with PCOS depend on the subtype of PCOS and are worse in the hyperandrogenic subtype. In women with thyroid dysfunction, TSH and thyroid hormone serum levels are known to influence lipid profiles or obesity risk. We think that further studies should take this into account, as well as analyzing the impact of AITD alone and dividing PCOS women into PCOS subgroups, as the heterogeneity of PCOS may confound the results.

Zhao H. et al. indicated that PCOS-AITD women had statistically significant higher serum insulin levels in 30 min and 60 min oral glucose tolerance test as well as higher insulin resistance indexes compared to PCOS women [14] All participants in the study were euthyroid and only the effect of aTPO and aTG level variations on metabolic state was investigated—as in our research. However, the limitation of the study is the small group size. Compared to the authors in our study, we did not find a statistically significant difference in serum glucose and insulin concentrations in the oral glucose tolerance test or in the HOMA-IR ratio between the PCOS and PCOS-AITD groups. However, all values were higher in the PCOS-AITD group.

Also, Kim JJ. et al. 2022 [21] also showed in their study that patients in the PCOS-AITD group had significantly higher body mass index, waist circumference, and homeostasis model assessment for insulin resistance levels than those without AITD.

Cenlin J. et al. [15] showed that PCOS-AITD women had higher serum fasting insulin levels, 60 min and 120 min oral glucose tolerance test insulin levels, and HOMA-IR compared to the PCOS group. In addition, the authors indicated a positive correlation between TG-Ab/TPO-Ab and fasting insulin and 60 min OGTT insulin (*p* < 0.05) [15]. It is known that the participants in this study were euthyroid, but the authors did not mention whether the participants used levothyroxine supplementation or were in the euthyroid phase of Hashimoto’s disease.

There are studies that suggest that thyroid dysfunction, understood as hypothyroidism, leads to an increased risk of type 2 diabetes [48,49]. It was indicated that among patients with type 2 diabetes, the prevalence of hypothyroidism was higher compared to patients without diabetes. It was also shown that patients with type 2 diabetes and hypothyroidism are at higher risk of diabetes complications than those without thyroid dysfunction [48]. On the other hand, research involving women with PCOS showed that the prevalence of diabetes was higher in the PCOS group compared with the control group. The prevalence of hypothyroidism was also higher in the PCOS group. According to the authors, this suggests that the presence of hypothyroidism in PCOS women may predispose them to diabetes more than PCOS alone [49].

However, the impact of elevated autoantibodies in euthyroid PCOS women on carbon metabolism remains unclear.

There are studies in the literature that confirm the effect of hypothyroidism on the lipid profile in patients with PCOS. As a result of the coexistence of the two conditions, higher levels of LDL cholesterol and triglycerides are observed when HDL cholesterol levels are reduced [47,50]. Interestingly, some authors suggest that elevated autoantibodies alone also increase the risk of lipid profile abnormalities in PCOS women, which is consistent with our findings [51,52]. In our study, we showed that PCOS-AITD women had statistically significantly higher serum concentrations of total cholesterol, LDL cholesterol, and triglycerides compared to the PCOS group.

Cenlin J. et al. [15] show that PCOS-AITD women had higher serum total cholesterol levels compared to PCOS women. In the aforementioned study, there were no differences in serum HDL cholesterol, LDL cholesterol, and triglyceride levels in the PCOS group vs. PCOS-AITD group [15]. As mentioned above, we do not know whether the participants are taking levothyroxine supplementation or are in a euthyroid state of disease.

On the other hand, according to Shokoufeh B. et al., there is no significant difference in lipid profile between PCOS vs. PCOS with hypothyroidism group except HDL cholesterol, which is higher in the PCOS group [53]. It is worth noting that the study groups in this research were small—41 women with PCOS and 41 women in the control group. To make a comparative analysis between the PCOS group and the PCOS-hypothyroidism group, only patients from the PCOS group (41 women) were considered. This study number is the major limitation of the research. In comparison with Shokoufeh B et al., in our study, we only included euthyroid patients and investigated the effect of elevated thyroid autoantibodies alone.

## 5. Conclusions

Polycystic ovary syndrome and autoimmune thyroid disease have a very high prevalence in women of reproductive age. It is common for the diseases to co-exist. There are also hypotheses of a common etiology and a common possible pathogenesis of these health conditions. Both PCOS and AITD with hypothyroidism can have common clinical manifestations, and both can have long-term negative health consequences, as well as a negative impact on life satisfaction and mental health. These facts mean that PCOS and AITD are health conditions that need to be managed by doctors of different specialties—from general practitioners, gynecologists, internists, diabetologists, dermatologists, endocrinologists to psychiatrists.

In our study, we have shown that the co-occurrence of autoimmunity and chronic low-grade inflammation, such as elevated thyroid autoantibodies in euthyroid PCOS women, is an additional risk factor for worsening the metabolic condition of PCOS patients by increasing BMI and worsening lipid profile. PCOS women without elevated thyroid autoantibodies have better lipid profiles and lower BMI values. This leads to the conclusion that thyroid autoantibodies may be an independent risk factor for worsening of negative metabolic consequences in PCOS women. 

All of these metabolic changes can increase the cardiovascular risk in these patients. Therefore, we believe that all women with PCOS should be tested for the presence of elevated thyroid autoantibodies, even if they are euthyroid. In addition, women with PCOS-AITD need special attention to maintain an appropriate body mass index and to prevent diabetes and cardiovascular disease. As there are only a few studies to date that have assessed the impact of thyroid autoantibodies alone on long-term health outcomes in women with PCOS, and the results of these are inconsistent, we believe that new studies are needed on this topic in a large group of patients. A potential limitation of the study is the homogeneity of the group—the study was conducted on a sample of the Polish population at a single academic center and was limited to women who voluntarily agreed to participate. To confirm the obtained results, similar or larger studies should be conducted on patient populations in other academic centers as well as among individuals of different nationalities.

## Figures and Tables

**Table 1:** Clinical and biochemical parameters of PCOS group and euthyroid PCOS-AITD group.

Parameter	PCOS Group (n = 230)	PCOS-AITD Group (n = 194)	*p*-Values	Effect Size (d Cohen’s)
**Age** (years)	25.22 (SD = 4.83)	28.02 (SD = 6.26)	<0.001 *	**−0.714**
**BMI** (kg/m^2^)	25.46 (SD = 5.54)	27.55 (SD = 7.71)	0.003 *	**−0.319**
**TSH** (µIU/mL)	1.62 (SD = 0.66)	2.12 (SD = 2.62)	0.010 *	**−0.272**
FT4 (pmol/mL)	12.37 (SD = 1.51)	12.68 (SD = 1.83)	0.054	-
**TPO-Ab** (IU/mL)	0.48 (SD = 0.51)	289.19 (SD = 510.18)	<0.001 *	**−0.837**
**TG-Ab** (IU/mL)	1.7 (SD = 1.29)	140.73 (SD = 643.7)	0.003 *	**−0.319**
Glucose 0′ (OGTT) (mg/dL)	91.98 (SD = 53.18)	89.49 (SD = 8.05)	0.519	-
Glucose 120′ (OGTT) (mg/dL)	116.97 (SD = 32.79)	116.28 (SD = 37.99)	0.844	-
Insulin 0′ (OGTT) (µU/L)	7.71 (SD = 4.86)	8.16 (SD = 4.55)	0.332	-
Insulin 60′ (OGTT) (µU/L)	61.96 (SD = 38.82)	69.55 (SD = 48.34)	0.085	-
Insulin 120′ (OGTT) (µU/L)	62.06 (SD = 60.57)	66.42 (SD = 52.44)	0.442	-
HOMA-IR	1.7 (SD = 1.21)	1.82 (SD = 1.09)	0.259	-
**Total cholesterol** (mg/dL)	180.16 (SD = 33.05)	189.57 (SD = 34.85)	0.005 *	**−0.278**
**LDL cholesterol** (mg/dL)	102.01 (SD = 31.03)	109.8 (SD = 29.9)	0.009 *	**−0.256**
HDL cholesterol (mg/dL)	58.2 (SD = 13.9)	59.51 (SD = 19.89)	0.440	-
**TG** (mg/dL)	96.82 (SD = 46.35)	107.77 (SD = 54.00)	0.027 *	**−0.219**

*T*-test; * < 0.05. Bold indicates statistically significant values.

**Table 2:** Relationship between autoimmunity and BMI.

**BMI (kg/m^2^)**	**PCOS Group** **(n = 230)**	**PCOS-AITD Group** **(n = 194)**	Chi-square = 7.956 *p* = 0.047 *
<18.5	4.5%	4.7%	
18.5–24.9	51.8%	40.2%	
25–29.9	23%	22.5%	
>30	20.7%	32.5%	

* < 0.05.

**Table 3:** Correlations between autoimmunity and metabolic parameters.

Parameter	TPO-Ab		TG-Ab	
	Rho	*p*-Value	Rho	*p*-Value
**BMI**	**0.122**	0.016 *	**0.159**	0.002 *
**Glucose 0′ (OGTT)**	0.058	0.232	**0.137**	0.005 *
Glucose 120′ (OGTT)	0.085	0.084	0.001	0.984
**Insulin 0′ (OGTT)**	**0.171**	<0.001 *	**0.110**	0.023 *
Insulin 60′ (OGTT)	0.088	0.074	0.078	0.116
**Insulin 120′ (OGTT)**	**0.180**	<0.001 *	0.082	0.099
**HOMA-IR**	**0.170**	<0.001 *	**0.134**	0.006 *
**Total cholesterol**	0.031	0.521	**0.141**	0.004 *
**LDL cholesterol**	0.058	0.237	**0.143**	0.003 *
HDL cholesterol	−0.089	0.067	−0.023	0.634
**TG**	0.092	0.059	**0.119**	0.015 *

* < 0.05. Bold indicates statistically significant values.

**Table 4:** Mini-review of research from years 2013–2024 on co-occurrence of AITD among PCOS women.

Publication	Study Group	Aim of the Study	Conclusions
Du D. and Li X. 2013 [19] (Meta-analysis)	726 PCOS women vs. 879 non-PCOS women	To investigate the relationship between PCOS and AITD.	The prevalence of AITD among PCOS women is significantly higher. PCOS may be a kind of autoimmune disease and has close association with AITD.
Duran C. et al. 2015 [24] (Case–control)	73 PCOS women vs. 60 controls	To investigate frequency of AITD and nodular goiter in patients with PCOS.	There is similar ratio of AITD women in both groups.
Karaköse M. et al. 2017 [23] (Case–control)	97 PCOS women vs. 71 controls	To detect the prevalence of AITD and nodular goiter in patients with PCOS.	The frequency of AITD and nodular goiter is significantly higher in the PCOS group.
Romitti M. et al. 2018 [6] (Systematic review and meta-analysis)	1210 PCOS women vs. 987 non-PCOS women	To evaluate the risk of AITD co-occurrence in PCOS women.	A significant association and higher prevalence is demonstrated between PCOS and AITD. Screening for thyroid function and thyroid autoantibodies should be considered even in absence of symptoms.
Ho CW. et al. 2020 [20] (Cohort)	6731 women with AITD vs. 26924 controls	To assess the prevalence of PCOS and it’s comorbidities in women with AITD.	The risk of PCOS and it’s comorbidities is higher in AITD women. There is a common link between two diseases.
Kim JJ. et al. 2022 [21] (Case–control)	210 PCOS women vs. 343 non-PCOS women	To assess the prevalence of AITD (TPO-Ab presence and specific thyroid USG signs) in PCOS women in comparison to healthy controls.	There is no difference in AITD prevalence among both groups. PCOS-AITD women have higher adiposity and insulin resistance index than PCOS women without AITD.
Sharma M. et al. 2022 [22] (Case–control)	33 PCOS women vs. 32 controls	To estimate association of anti-TPO with LH/FSH in PCOS women.	There is a high prevalence of AITD in euthyroid PCOS women.

**Table 5:** Mini-review of the literature from years 2013–2024 on the impact of AITD on metabolic consequences in PCOS women.

Publication	Study Group	Conclusions
Romitti M. et al. 2018 [6] (Systematic review and meta-analysis)	1210 PCOS women vs. 987 non-PCOS women	There is no difference in BMI between PCOS group and PCOS-AITD group There is no correlation between AITD and BMI in PCOS women Co-occurrence of PCOS and subclinical hypothyroidism correlate with: - mild alterations in serum lipids, - mild alterations in HOMA-IR. Impact of AITD alone was not assessed.
Ulrich J. et al. 2018 [17] (Retrospective cohort)	827 PCOS women	PCOS-AITD women had higher BMI in comparison to PCOS group. PCOS-AITD group had lower prevalence of elevated testosterone, free androgen index and hyperandrogenemia. PCOS-AITD group had lower testosterone serum concentration vs. PCOS group.
Ho CW. et al. 2020 [20] (Cohort)	6731 women with AITD vs. 26924 controls	Prevalence of diabetes was higher in PCOS-AITD group with odds ratio 2.48 Prevalence of hyperlipidemia was higher in PCOS-AITD group with odds ratio 2.05 Prevalence of Coronary Artery Disease was higher in PCOS-AITD group with odds ratio 2.63
Zhao H. et al. 2021 [14] (Comparative)	52 PCOS-AITD women vs. 112 PCOS women	PCOS-AITD group had higher insulin serum levels in 30 min and 60 min of oral glucose tolerance test vs. PCOS group PCOS-AITD group had higher insulin resistance index vs. PCOS group PCOS-AITD group had lower serum levels of free thyroxine and thyrotropin and higher ratio of free thyroxine to thyrotropin vs. PCOS group
Kim JJ. et al. 2022 [21] (Case–control)	210 PCOS women vs. 343 non-PCOS women	PCOS-AITD group in comparison to PCOS group presented significantly higher: - body mass indexes, - waist circumferences (adiposity), - homeostasis model assessment for insulin resistance.
Cenlin J. et al. 2023 [15] (Cross-sectional)	164 PCOS women vs. 49 PCOS-AITD women	There was no difference in body mass index among PCOS vs. PCOS-AITD group PCOS-AITD group had higher fasting insulin serum level as well as a higher insulin serum level in 60 min and 120 min of oral glucose tolerance test vs. PCOS group. PCOS-AITD group had higher HOMA-IR vs. PCOS group. PCOS-AITD group had higher level of total cholesterol vs. PCOS group. There were no differences in HDL-cholesterol, LDL-cholesterol and triglycerides serum levels among PCOS vs. PCOS-AITD group. PCOS-AITD group had higher TSH serum level and lower free thyroxine and thyrotropin serum level vs. PCOS group. In PCOS-AITD group there was a positive correlation between TG-Ab/TPO-Ab and fasting insulin and 60 min OGTT insulin. (*p* < 0.05). There was also positive correlation between TG-Ab/TPO-Ab and insulin in 120 min of OGTT, insulin resistance and total cholesterol but it was not statistically significant.

## Data Availability

The data presented in this study are available on request from the corresponding author. The data are not publicly available due to current calculations to the next publication from this study.

## Supplementary Materials

The following supporting information can be downloaded at: https://www.mdpi.com/article/10.3390/metabo15030141/s1, Table S1: Ranges of the hormonal and biochemical parameters standard concentrations.
