# Supplementary material for: Autoimmune Processes and Chronic Inflammation as Independent Risk Factors for Metabolic Complications in Women with Polycystic Ovary Syndrome
Source: Metabolites. 2025 Feb 20;15(3):141. doi: 10.3390/metabo15030141 (PMC11943895; doi:10.3390/metabo15030141)
Supplement: Supplementary file 1 [file metabolites-15-00141-s001.zip › metabolites-3465175-supplementary.pdf]

1. Supplementary materials:

Table S1. Ranges of the hormonal and biochemical parameters standard concentrations.

	PARAMETER	Unit	Range of standard concentration
TSH	thyroid-stimulating hormone	μIU/ml	0.35-4.94
FT4	free thyroxine	pmol/ml	age 11-21: 10.04-17.25;<br>age 21-45: 9.0-19.05
TPO-Ab	anti-thyroid peroxidase	IU/ml	<5.61
TG-Ab	anti-thyroglobulin	IU/ml	<4.1
Total Chol	Total cholesterol	mg/dl	<190 – optimal concentration<br>191-239 – limit concentration<br>>240 – high concentration
LDL Chol	low-density lipoprotein cholesterol	mg/dl	<115
HDL Chol	high-density lipoprotein cholesterol	mg/dl	<45 – low concentration<br>46-60 – medium concentration<br>>60 – high concentration
TG	triglycerides	mg/dl	<150 – optimal concentration<br>150-199 – limit concentration<br>200-499 – high concentration<br>>500 – extremely high concentration
GLU 0′ (OGTT)	fasting glucose in oral glucose tolerance test	mg/dl	70-99
GLU 120′ (OGTT)	glucose at 120 min of oral glucose tolerance test	mg/dl	<140 – optimal<br>140-199 – impaired glucose tolerance<br>>200 - diabetes
INS 0′ (OGTT)	fasting insulin in oral glucose tolerance test	μU/l	<10
HOMA-IR	homeostatic model assessment for insulin resistance	-	<2
